# GWAS on family history of Alzheimer’s disease

**DOI:** 10.1038/s41398-018-0150-6

**Published:** 2018-05-18

**Authors:** Riccardo E. Marioni, Sarah E. Harris, Qian Zhang, Allan F. McRae, Saskia P. Hagenaars, W. David Hill, Gail Davies, Craig W. Ritchie, Catharine R. Gale, John M. Starr, Alison M. Goate, David J. Porteous, Jian Yang, Kathryn L. Evans, Ian J. Deary, Naomi R. Wray, Peter M. Visscher

**Affiliations:** 1 Centre for Genomic and Experimental Medicine, Institute of Genetics and Molecular Medicine, University of Edinburgh, Edinburgh, EH4 2XU UK; 2 Centre for Cognitive Ageing and Cognitive Epidemiology, University of Edinburgh, Edinburgh, EH8 9JZ UK; 3 Institute for Molecular Bioscience, University of Queensland, Brisbane, QLD 4072 Australia; 4 Social, Genetic and Developmental Psychiatry Centre, Institute of Psychiatry, Psychology & Neuroscience, King’s College London, London, SE5 8AF UK; 5 Department of Psychology, University of Edinburgh, Edinburgh, EH8 9JZ UK; 6 Centre for Dementia Prevention, Centre for Clinical Brain Sciences, University of Edinburgh, Edinburgh, EH8 9YL UK; 7 MRC Lifecourse Epidemiology Unit, University of Southampton, Southampton, SO16 6YD UK; 8 Alzheimer Scotland Dementia Research Centre, University of Edinburgh, Edinburgh, EH8 9JZ UK; 9 Departments of Neuroscience, Neurology and Genetics and Genomic Sciences, Ronald M. Loeb Center for Alzheimer’s disease, Icahn School of Medicine at Mount Sinai, New York, NY 10029-5674 USA; 10 Queensland Brain Institute, University of Queensland, Brisbane, QLD 4072 Australia

**Keywords:** Genetics, Neuroscience

## Abstract

Alzheimer’s disease (AD) is a public health priority for the 21st century. Risk reduction currently revolves around lifestyle changes with much research trying to elucidate the biological underpinnings. We show that self-report of parental history of Alzheimer’s dementia for case ascertainment in a genome-wide association study of 314,278 participants from UK Biobank (27,696 maternal cases, 14,338 paternal cases) is a valid proxy for an AD genetic study. After meta-analysing with published consortium data (*n* = 74,046 with 25,580 cases across the discovery and replication analyses), three new AD-associated loci (*P* < 5 × 10^−8^) are identified. These contain genes relevant for AD and neurodegeneration: *ADAM10*, *BCKDK/KAT8* and *ACE*. Novel gene-based loci include drug targets such as *VKORC1* (warfarin dose). We report evidence that the association of SNPs in the *TOMM40* gene with AD is potentially mediated by both gene expression and DNA methylation in the prefrontal cortex. However, it is likely that multiple variants are affecting the trait and gene methylation/expression. Our discovered loci may help to elucidate the biological mechanisms underlying AD and, as they contain genes that are drug targets for other diseases and disorders, warrant further exploration for potential precision medicine applications.

## Introduction

The genetic epidemiology of late-onset Alzheimer’s disease (LOAD) has advanced over the last decade^1^, with >20 independent loci associated with the disease in addition to *APOE*^2^. Presently, the largest meta-analytic genome-wide association study (GWAS) for LOAD employed a two-stage study design. First, 17,008 cases were compared to 37,154 controls. A total of 11,632 single-nucleotide polymorphisms (SNPs) with *P* < 1 × 10^−3^ from this meta-analysis were included in the second stage that compared 8,572 cases to 11,312 controls. A meta-analysis of the SNPs included in stages 1 and 2 was also performed^3^.

One difficulty in traditional studies of AD is case ascertainment^4^—either directly for prevalent cases or indirectly through prospective cohort studies for incident cases. A recent GWAS study on a subset of the UK Biobank cohort used information from family history (parent or first-degree relative with AD or dementia) as a proxy-phenotype for the participants^5^. When meta-analysed with the GWAS summary data highlighted above^3^, four new loci were identified.

The UK Biobank proxy-phenotype AD question, which is used here, does not incorporate biomarker data that are required for a clinical diagnosis. However, it is easy to administer at scale and we show that it has a near-unit genetic correlation with the AD results from the LOAD meta-analysis^3^, where many of the samples also lacked a confirmed diagnosis by biomarker levels or autopsy.

In the present study, we related proxy-phenotype information on dementia (i.e., reporting a parent with Alzheimer’s dementia or dementia) to genetic data from 314,278 individuals from the UK Biobank cohort to identify new AD-associated loci. This sample includes individuals from the previous proxy-phenotype AD study by Liu et al.^5^ GWA studies were conducted separately for maternal and paternal AD due to a 1.7-fold difference in disease prevalence—9.6% and 5.5%, respectively. The summary statistics from these models were meta-analysed with those from the largest publicly available case–control study^3^. Sensitivity analyses showed that an overlap of controls in the maternal and paternal GWAS did not bias the results. Genetic correlation analysis showed the self-reported measure of parental AD to be an accurate proxy for clinical diagnosis, validating the global meta-analysis. In addition, we tested for causal evidence of our SNP–AD associations being mediated through gene expression and DNA methylation in the prefrontal cortex.

## Subjects and methods

### UK Biobank cohort

UK Biobank data^6^ (http://www.ukbiobank.ac.uk) were collected on over 500,000 individuals aged between 37 and 73 years from across Great Britain (England, Wales and Scotland) at the study baseline (2006–2010), including health, cognitive and genetic data.

The Research Ethics Committee (REC) granted ethical approval for the study—reference 11/NW/0382—and the current analysis was conducted under data application 10,279.

### Genotyping

Genotyping details for the UK Biobank cohort have been reported previously^7,8^. Briefly, two custom genotyping arrays were utilised with 49,950 participants typed using the UK BiLEVE Axiom Array and 438,427 participants typed using the UK Biobank Axiom Array^7,8^. The released genotyped data contained 805,426 markers on 488,377 individuals. Imputed genotypes were supplied with the UK Biobank data with the Haplotype Reference Consortium (HRC) used as the imputation reference panel^7^.

Downstream quality control steps conducted for the current analysis included removing (1) those with non-British ancestry based on both self-report and a principal components analysis, (2) outliers based on heterozygosity and missingness, (3) individuals with sex chromosome configurations that were neither XX nor XY, (4) individuals whose reported sex did not match inferred sex from their genetic data and (5) individuals with >10 putative third-degree relatives from the kinship table. This left a sample of 408,095 individuals. To remove the possibility of double contributions from sibs, whose parents will have the same AD status, we first considered a list of all participants with a relative (*N* = 131,790). A genetic relationship matrix was built for these individuals using GCTA-GRM^9^ and a relationship threshold of 0.025 was applied to exclude related individuals. After removing one person from each pair of related individuals, the sample size was 332,050. Quality control thresholds applied to the GWAS included: minor allele frequency >0.01, imputation quality score >0.3 and restriction to HRC-imputed SNPs, leaving a total of 7,795,605 SNPs for the GWAS.

### Phenotypes

Family history of Alzheimer’s disease was ascertained via self-report. Participants were asked “Has/did your father ever suffer from Alzheimer’s disease/dementia?” and “Has/did your mother ever suffer from Alzheimer’s disease/dementia?” Self-report data from the initial assessment visit (2006–2010), the first repeat assessment visit (2012–2013) and the imaging visit (2014+) were aggregated with exclusions made for participants whose parents were: aged under 60 years; dead before reaching age 60 years; without age information. After merging with the genetic data, this left 27,696 cases of maternal AD with 260,980 controls, and 14,338 cases of paternal AD with 245,941 controls. There were 314,278 instances where AD information was available on at least one parent. Given the expected difference in disease prevalence due to sex differences in longevity—AD prevalence was 1.7-fold higher in mothers compared to fathers—GWA studies were performed separately for maternal and paternal AD.

### Genome-wide association study

The GWA studies were conducted using BGENIE^7^. The outcome variable was the residuals from a linear regression model of maternal or paternal AD on age of parent at death or at time of the offspring’s self-report, assessment centre, genotype batch, array and 40 genetic principal components. The predictor variable was the autosomal SNP and an additive model was considered.

The GWAS linear regression coefficients were converted to odds ratios using observed sample prevalences of 0.096 and 0.055 for maternal and paternal AD, respectively^10^. Subsequently, the log-odds were multiplied by two so that the effect sizes are reported on the same scale as a traditional case–control design^5^. Briefly, the conversion to odds ratios uses the following equation, derived in Lloyd-Jones et al.^10^, where *k* = disease prevalence, *p* = population allele frequency and *β* = the estimated SNP regression coefficient on the binary disease scale from the GWAS: ${\mathrm{OR}} = \frac{{\left( {{{k}} + {{\beta }}\left( {1 - {{p}}} \right)} \right) \times \left( {1 - {{k}} + {{\beta p}}} \right)}}{{\left( {{{k}} - {{\beta p}}} \right) \times \left( {1 - {{k}} - {{\beta }}\left( {1 - {{p}}} \right)} \right)}}$. SEs for the log-odds were then calculated based on the adjusted OR and the *P*-value from the initial GWAS (Supplementary Note 1). The ORs and SEs were then carried forward to a SE-weighted meta-analysis in METAL^11^, first to create a UK Biobank parental meta-analysis, and then with the stage 2 summary output from the International Genomics of Alzheimer’s Project (IGAP) study^3^ and the stage 1 output for the SNPs that did not contribute to stage 2. Linkage disequilibrium score (LDSC) regression was used to estimate the genetic correlation between the maternal and paternal AD GWAS results and to test for residual confounding in the meta-analysis by examining the LDSC intercept^12,13^.

The number of independent loci from the meta-analysis was determined by using the default settings in FUMA^14^. Independent lead SNPs had *P* < 5 × 10^−8^ and were independent at *r*^2^ < 0.6. Within this pool of independent SNPs, lead SNPs were defined as those in LD at *r*^2^ < 0.1. Loci were defined by combining lead SNPs within a 250 kb window and all SNPs in LD of at least *r*^2^ = 0.6 with one of the independent SNPs. A gene-based analysis was carried out on all SNP output using the MAGMA software^15^ with default settings (SNP-wise (mean) model for each gene), and assuming a constant sample size for all genes. A Bonferroni-adjusted *P*-value of 0.05/18,251 = 2.7 × 10^−6^ was used to identify significant genes. The 1000 genomes phase 3 data^16^ were used to map LD in both the independent locus and MAGMA analyses.

### Summary data-based Mendelian randomisation

To test for pleiotropic associations between SNPs and AD and gene expression/DNA methylation in the brain, summary data-based Mendelian randomisation (SMR) was performed^17^. GWAS summary output from the meta-analysis of UK Biobank and IGAP (sample size specified as 314,278 + 74,046 = 388,324) were included along with expression Quantitative Trait Loci (QTL) summary output from the Common Mind Consortium, which contains data on >600 dorsolateral prefrontal cortex samples, and DNA methylation QTL summary output on 258 prefrontal cortex samples (age >13)^18^. The reference genotypes were based on the Health and Retirement Study, imputed to the 1000 Genomes phase 1 reference panel. SNP exclusions included: imputation score <0.3, Hardy–Weinberg *P*-value < 1 × 10^−6^ and a minor allele frequency <0.01. Related individuals, based on a genomic-relationship matrix cutoff of 0.05, were removed. Two sets of eQTL summary data were considered (1) after adjustment for diagnosis, institution, sex, age of death, post-mortem interval, RNA integrity number (RIN), RIN^2^, and clustered library batch (2) with additional adjustments for 20 surrogate variables to account for additional possible confounders. Five ancestry vectors were included as covariates in the eQTL analyses. Further details are available at: https://www.synapse.org/#!Synapse:syn4622659. Default parameters for the SMR analysis were used and *cis* eQTLs/methQTLs were considered for analysis. Bonferroni-corrected *P*-value thresholds were applied (*P* < 0.05/2011 = 2.5 × 10^−5^ for eQTL data set 1, *P* < 0.05/4380 = 1.1 × 10^−5^ for eQTL data set 2 and *P* < 0.05/54,624 = 9.2 × 10^−7^ for the methQTL data set). The SMR *P*-value highlights candidate transcripts or methylation sites through which a *cis* SNP may be acting on the outcome, AD. The heterogeneity in dependent instruments (HEIDI) *P*-value indicates evidence for a single causal SNP (effect on AD is mediated through the transcript/methylation site if *P* > 0.05) or different SNPs affecting AD and the transcript/methylation site if *P* < 0.05.

## Results

### UK Biobank GWAS

There were 27,696 cases of maternal AD (260,980 controls, prevalence of 9.6%) and 14,338 cases of paternal AD (245,941 controls, prevalence of 5.5%) in the UK Biobank. The genetic correlation between maternal and paternal AD was not significantly different from unity (*r*_g_ = 0.65, SE 0.59), although the SE is large. Both traits had a high genetic correlation with the case–control summary output from the IGAP study: *r*_g_ with maternal and paternal AD was 0.91 (SE 0.24) and 0.67 (0.40), respectively, both not significantly different from unity but with large SEs.

Prior to meta-analysing the UK Biobank parental summary statistics with the IGAP output, we investigated the influence of overlapping proxy controls in UK Biobank. The *P*-values from a single GWAS of parental AD status (0, 1 or 2 parents with AD) correlated 0.99 with those from a meta-analysis of separate maternal AD and paternal AD; the regression of −log10 *P*-values on each other gave an intercept of 0 and a slope of 1. A meta-analysis of the summary statistics from the maternal and paternal results is therefore essentially equivalent to the analysis of parental AD status. The linear regression effect sizes from the GWAS were converted to odds ratios prior to the meta-analysis^10^.

### UK Biobank paternal and maternal meta-analysis

Six genome-wide significant loci were identified in the UK Biobank paternal and maternal GWAS meta-analysis (Supplementary Table 1, GWAS summary statistics available at www.ccace.ed.ac.uk/node/335). All were located in established AD loci. The top lead SNPs were rs679515 (*P* = 5.2 × 10^−9^, chr1, *CR1* locus), rs6733839 (*P* = 1.1 × 10^−27^, chr2, *BIN1* locus), rs7384878 (*P* = 1.3 × 10^−10^, chr7, *PILRA* locus), rs3851179 (*P* = 1.8 × 10^−12^, chr11, *PICALM* locus), rs3845261 (*P* = 4.0 × 10^−8^, chr17, *ZNF232* locus) and rs10119 (*P* = 3.5 × 10^−308^, chr19, *APOE/TOMM40* locus). The chromosome 17 locus is ~100 kb from the *SCIMP* gene-variant identified by Liu et al.^5^

### UK Biobank parental and IGAP meta-analysis

**Fig. 1:**
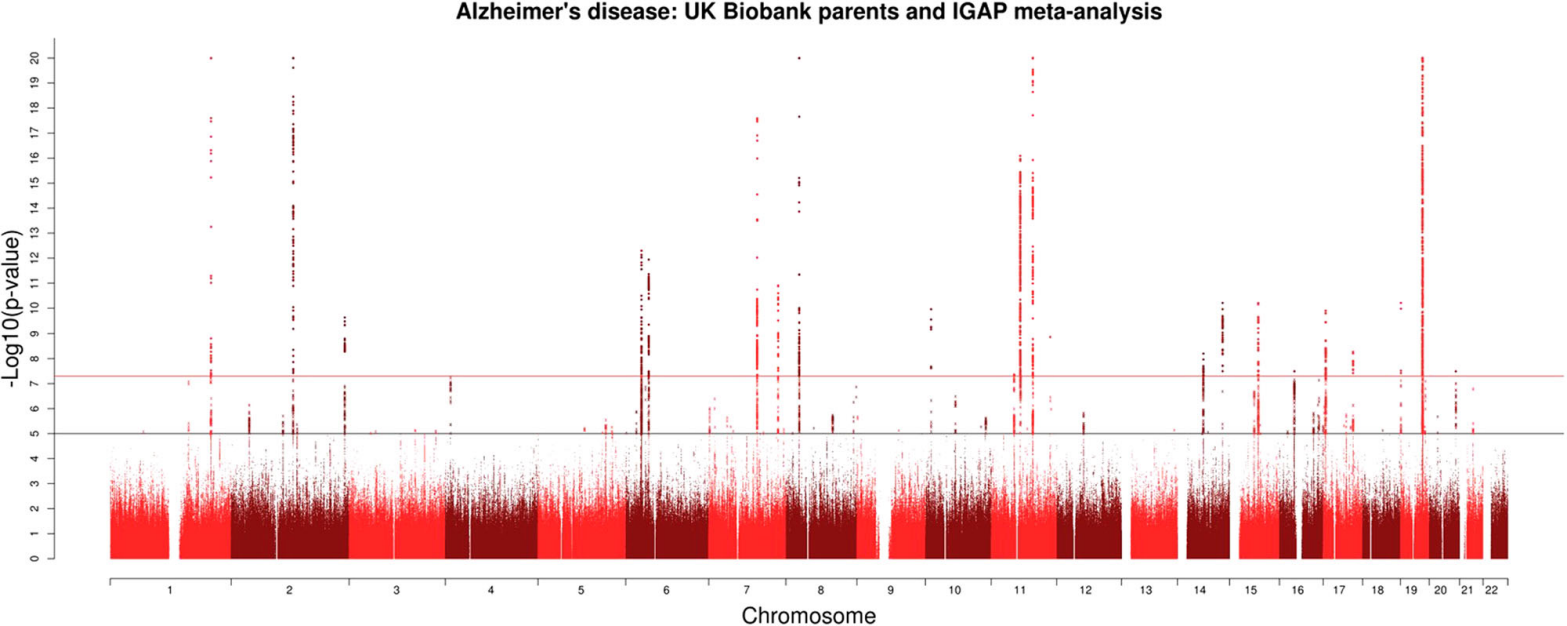
Manhattan plot for the meta-analysis of maternal and paternal Alzheimer’s disease in UK Biobank and the results from stage 1 and stage 2 of IGAP^3^. The red line indicates *P* = 5 × 10^−8^ and the blue line indicates *P* = 1 × 10^−5^. *P*-values truncated at 1 × 10^−20^

The meta-analysis of the maternal and paternal AD history in UK Biobank with the IGAP data identified 83 lead SNPs and 264 independent significant SNPs with *P* < 5 × 10^−8^ from 26 genomic risk loci. The majority (*n* = 45) of the lead SNPs were located in the gene-dense *APOE*/*TOMM40* locus on chromosome 19 (Fig. 1 and Supplementary Tables 2 and 3; GWAS summary statistics are available for the out-meta-analysed SNPs online: www.ccace.ed.ac.uk/node/335). The LDSC regression intercept term from the meta-analysis summary output was 1.029 (SE 0.0097), and around 81% of the genomic inflation (*λ* = 1.11) was due to the polygenic signal, rather than sources of confounding including population stratification and cryptic relatedness.

### Novel genome-wide significant loci

Of the 26 significant risk loci, seven were novel. These include the three loci highlighted in the previous version of the manuscript (Table 1 and Supplementary Figures 1–3). The top lead SNPs were: rs593742 (*ADAM10*, locus chr15:58873555–59132827); rs889555 210 (*BCKDK/KAT8*, locus chr16:30916129–31155458); and rs138190086 (*ACE*, locus 211 chr17:61001497–61559625).

**Table 1:** Novel SNPs (*P* < 5 × 10^−8^) from the meta-analysis of UK Biobank parental history of Alzheimer’s disease with results from IGAP

Locus	Chr	Lead SNP	A1	A2	BP	Freq	Locus start	Locus end	OR 95% CI	*P*	Direction^a^
1	15	rs593742	A	G	59045774	0.69	58873555	59120077	1.06 (1.04, 1.07)	6.20*E*−11	++?+
2	16	rs889555	T	C	31122571	0.29	30916129	31155458	0.95 (0.94, 0.97)	3.20*E*−08	−−?−
3	17	rs6504163	T	C	61545779	0.63	61545779	61578207	1.05 (1.03, 1.07)	5.40*E*−09	+++?

*Chr* chromosome, *BP* base pair, *Freq* frequency of allele A1, *OR* odds ratio and 95% confidence interval

^a^UK Biobank maternal AD, UK Biobank paternal AD, IGAP stage 1, IGAP stage 2

We now report four additional genome-wide significant loci: rs9381040 (*TREML2*, locus chr6: 41031273-41164788); rs59685680 (*SPPL2A*, locus chr15:50716633-51060127); rs4985556 (*IL-34*, locus chr16:70638035-70694991); rs12444183 (PLCG2, locus chr16:81773003-81773816).

### Replication of IGAP SNPs

Seventeen of the 21 previously reported SNPs^3^ associated with AD were genome-wide significant (*P* < 5 × 10^−8^) in the current meta-analysis, with two other SNPs (rs2718058 and, rs10838725) having *P* < 1 × 10^−7^ (Supplementary Table 4). The *MEF2C* variant, rs190982, had a meta-analysis *P*-value of 3.7 × 10^−4^ and rs8093731 (a *DSG2* variant), which was genome-wide significant in stage 1 but not stage 2 of IGAP, had a meta-analysis *P*-value of 7.2 × 10^−4^. There was complete sign-concordance between UK Biobank and IGAP with the exception of rs190982 (Supplementary Table 4). The odds ratios between the maternal and paternal analysis for the top 21 IGAP SNPs were correlated *r* = 0.76. Both also correlated highly with the effect sizes reported in the IGAP analysis (*r* = 0.89 and 0.85, respectively).

### Gene-based analysis

A total of 87 genes were significant at a Bonferroni threshold of *P* < 2.7 × 10^−6^ (Supplementary Table 5). Gene ontology analysis, using a Fisher exact test to compare the number of significant genes in each gene set with the expected number, showed significant enrichment for 51 gene sets, including those linked to the regulation of amyloid-beta, neurofibrillary tangle assembly, immune response, and cholesterol and lipid transport (Supplementary Table 6).

### Summary data-based Mendelian randomisation

Pleiotropic associations between AD and gene expression in the brain were tested using SMR^17^. GWAS summary data for AD were taken from the UK Biobank and IGAP meta-analysis. eQTL summary data came from the Common Mind Consortium (*n* > 600 dorsolateral prefrontal cortex samples: data set 1 adjusted for age at death, sex and institution; data set 2 made additional adjustments for 20 surrogate variables). MethQTL data came from 258 dorsolateral prefrontal cortex samples (participants aged 13 years and older—adjustments were made for the first 5 genetic MDS components and first 11 methylation PCs)^18^. We found evidence of brain expression and DNA methylation associated with AD in the *TOMM40* gene (part of the *APOE*/*TOMM40* cluster on chromosome 19) in both the second eQTL model and also in the methQTL model (Supplementary Tables 7–9). However, the HEIDI *P*-values were <0.05 for both analyses, indicating that the associations were unlikely to be driven by a single causal variant affecting both expression/methylation and AD.

Different top QTL SNPs in *TOMM40* were identified in the two analyses: rs760136 and rs7259620 although they were in perfect LD in European samples^19,20^ (*R*^2^ = 1.00). The SNPs have differing LD patterns with the *APOE* allele defining SNPs, rs7412 (*R*^2^ = 0.001, *D*′ = 0.03) and rs429358 (*R*^2^ = 0.15, *D*′ = 1.0). A significant SMR association with HEIDI *P*-value >0.05, indicating pleiotropy, was observed for *KAT8* and *CR1* in the second eQTL analysis (Supplementary Table 8 and Supplementary Figures 4 and 5) and for *STAG3* (chr7, *PILRA* locus), *CD2AP* (chr6), *HLA-DRB1* (chr6, locus not reported in Lambert et al.^3^), and *PSMC3* (chr11, *CELF* locus) in the methQTL analysis (Supplementary Table 9 and Supplementary Figures 6–9).

## Discussion

Using recently established proxy-phenotype methods for case ascertainment, we show this approach to be valid in GWAS of AD. Meta-analysing the proxy-phenotype summary statistics from UK Biobank with those from a case–control study (IGAP), we identified three new genome-wide significant loci that contain several relevant genes.

*ACE* determines levels of angiotensin II, which has trophic actions within the brain and contributes to the regulation of cerebral blood flow^21^. Previous meta-analyses of candidate gene studies identified variants within *ACE* to be associated with AD, though not at genome-wide significance^22,23^. *ACE* variants have also been linked to atrophy of the hippocampus and amygdala^24^, and CSF-ACE protein levels correlate with CSF tau and phosphorylated tau^25,26^.

*ADAM10* is involved in the cleavage of amyloid-beta precursor protein^27^, which is involved in the deposition of amyloid beta, a major neurological hallmark of AD. ADAM10 has been proposed as potential therapeutic agent in AD therapy^27,28^. Rare variants in *ADAM10* have also been linked to LOAD^29^.

The *BCKDK/KAT8* locus contains the *VKORC1* gene, which was a genome-wide significant gene-based finding (*P* = 5.1 × 10^−8^). The *VKORC1* variant, rs9923231, whose T allele was suggestively associated with an increased risk of AD (*P* = 1.8 × 10^−7^), is strongly associated with the need for a reduced dose of warfarin anticoagulation^30,31^. *KAT8* is regulated by *KANSL1*, which has been linked to AD as a genome-wide significant finding in *APOE* e4-negative individuals^32^.

Our cutoff for relatedness of 0.025 in UK Biobank was very stringent, in particular since disease status was on parents of genotyped individuals, so that correlations in proxy-phenotypes from relatives (e.g., cousins) are likely to be very small. When we used a more relaxed threshold of 0.4 (excluding first-degree relatives only), we increased our sample size to 385,869 and found three additional loci and top lead SNPs: a gene desert on chr4 (rs6448453, *P* = 2.8 × 10^−9^, chr4:11014822–11041549) that is ~400 kb from the *CLNK* gene; a region on chromosome 17 that includes the benzodiazepine receptor-associated protein 1 (*BZRAP1*) gene (rs2526378, *P* = 2.0 × 10^−8^, chr17:56398006-56410041) and a locus including variants in the amyloid precursor protein (*APP*) gene on chromosome 21 (rs4817090, *P* = 4.8 × 10^−8^, chr21:27454995-27534261). The corresponding *P*-values for these three lead SNPs in the GWAS meta-analysis with the more stringent relatedness cutoff were *P* = 9.6 × 10^−8^, 7.5 × 10^−7^, and 1.3 × 10^−7^, respectively. Two additional loci were reported as significant using the less stringent relatedness threshold in the previous version of the manuscript. Both were just below genome-wide significance in the updated analysis: rs4575098, *P* = 8.8 × 10^−8^, *ADAMTS4* and rs1171812, *P* = 6.4 × 10^−8^, *CCDC6*. Moreover, the novel *KAT8*/*BCKDK* locus, which is reported in the main results section, is not genome-wide significant in the analysis using the less stringent relatedness threshold (rs889555, *P* = 1.7 × 10^−7^).

The gene-set enrichment analysis implicated pathways involved in AD neuropathology (amyloid and neurofibrillary tangles) in addition to immune response, lipid and cholesterol metabolism, as previously reported in a gene-set analysis on the IGAP data^33^. A separate gene-set analysis on the meta-analysed paternal and maternal summary statistics from UK Biobank showed a highly overlapping set of enriched pathways (Supplementary Table 10).

An integrative analysis of eQTL and methQTL with the GWAS summary data identified one previously identified AD gene, *TOMM40*, as having its gene expression and methylation levels associated with AD. The most parsimonious explanation of these results is the existence of multiple causal variants, some affecting AD and others affecting expression or methylation. A previous SMR analysis of AD and LDL cholesterol identified evidence of 16 pleiotropic SNPs, 12 of which were located in the *APOE* region^34^. Given the involvement of immunity in the aetiology of AD, as highlighted by the pathway analysis, it may be relevant to consider immune cells, such as monocytes, for the SMR analysis.

The main strength of the study is the proxy-phenotype approach, which resulted in over 42,000 proxy cases for the GWAS analysis. However, the question used to determine parental AD status may have resulted in some responders being unable to discriminate Alzheimer’s disease and dementia from other dementia sub-types, which have different presentations and genetic architectures^35,36^. This method of proxy-case ascertainment may have influenced the loci uncovered. Parental dementia status is partly dependent on longevity, with age being the biggest risk factor for AD. We partially controlled for this by excluding participants whose parents were younger than or died prior to reaching the age of 60 years when AD incidence is extremely low. The misclassification of case status via incorrect informant reporting will have reduced the power to detect true effects.

## Conclusion

We identified three new AD-associated loci that have known and putative biological processes associated with Alzheimer’s disease. These findings help to elucidate the biological mechanisms underlying AD and, given that some loci (*VKORC1*, *ACE*) are existing drug targets for other diseases and disorders, warrant further exploration for potential precision medicine and clinical trial applications.

## Disclaimer

The funders had no role in study design, data collection and analysis, decision to publish or preparation of the manuscript.

## Supplementary information

Supplementary Figures

Supplementary Tables

Supplementary Note
