# Correction: GWAS on family history of Alzheimer’s disease

**DOI:** 10.1038/s41398-019-0498-2

**Published:** 2019-06-06

**Authors:** Riccardo E. Marioni, Sarah E. Harris, Qian Zhang, Allan F. McRae, Saskia P. Hagenaars, W. David Hill, Gail Davies, Craig W. Ritchie, Catharine R. Gale, John M. Starr, Alison M. Goate, David J. Porteous, Jian Yang, Kathryn L. Evans, Ian J. Deary, Naomi R. Wray, Peter M. Visscher

**Affiliations:** 1 Centre for Genomic and Experimental Medicine, Institute of Genetics and Molecular Medicine, University of Edinburgh, Edinburgh, EH4 2XU UK; 2 Centre for Cognitive Ageing and Cognitive Epidemiology, University of Edinburgh, Edinburgh, EH8 9JZ UK; 3 Institute for Molecular Bioscience, University of Queensland, Brisbane, QLD 4072 Australia; 4 Social, Genetic and Developmental Psychiatry Centre, Institute of Psychiatry, Psychology & Neuroscience, King’s College London, London, SE5 8AF UK; 5 Department of Psychology, University of Edinburgh, Edinburgh, EH8 9JZ UK; 6 Centre for Dementia Prevention, Centre for Clinical Brain Sciences, University of Edinburgh, Edinburgh, EH8 9YL UK; 7 MRC Lifecourse Epidemiology Unit, University of Southampton, Southampton, SO16 6YD UK; 8 Alzheimer Scotland Dementia Research Centre, University of Edinburgh, Edinburgh, EH8 9JZ UK; 9 Departments of Neuroscience, Neurology and Genetics and Genomic Sciences, Ronald M. Loeb Center for Alzheimer’s disease, Icahn School of Medicine at Mount Sinai, New York, NY 10029-5674 USA; 10 Queensland Brain Institute, University of Queensland, Brisbane, QLD 4072 Australia

**Correction to: Translational Psychiatry**

10.1038/s41398-018-0150-6 published online 18 May 2018

In the original Article, GWAS analysis of the UK Biobank data was based on standardised SNP dosages (standardised by their standard deviation) as opposed to raw SNP dosages. The standard error-weighted meta-analysis of the UK Biobank data with the IGAP findings was therefore incorrect. The meta-analysis has been re-run as previously specified (the key findings remain although four new genome-wide significant loci have been identified). There have also been updates to the Supplementary Tables and Fig. 1.

**Fig. 1:**
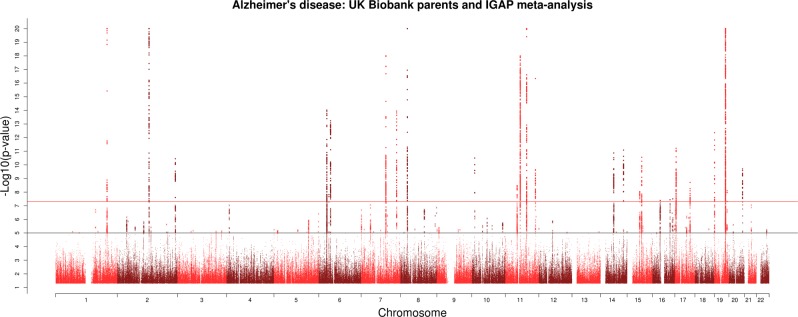
▓

