# Supplementary material for: GWAS on family history of Alzheimer’s disease
Source: Transl Psychiatry. 2018 May 18;8:99. doi: 10.1038/s41398-018-0150-6 (PMC5959890; doi:10.1038/s41398-018-0150-6)

**Figure S1.** Plot of genome-wide significant *ADAM10* locus


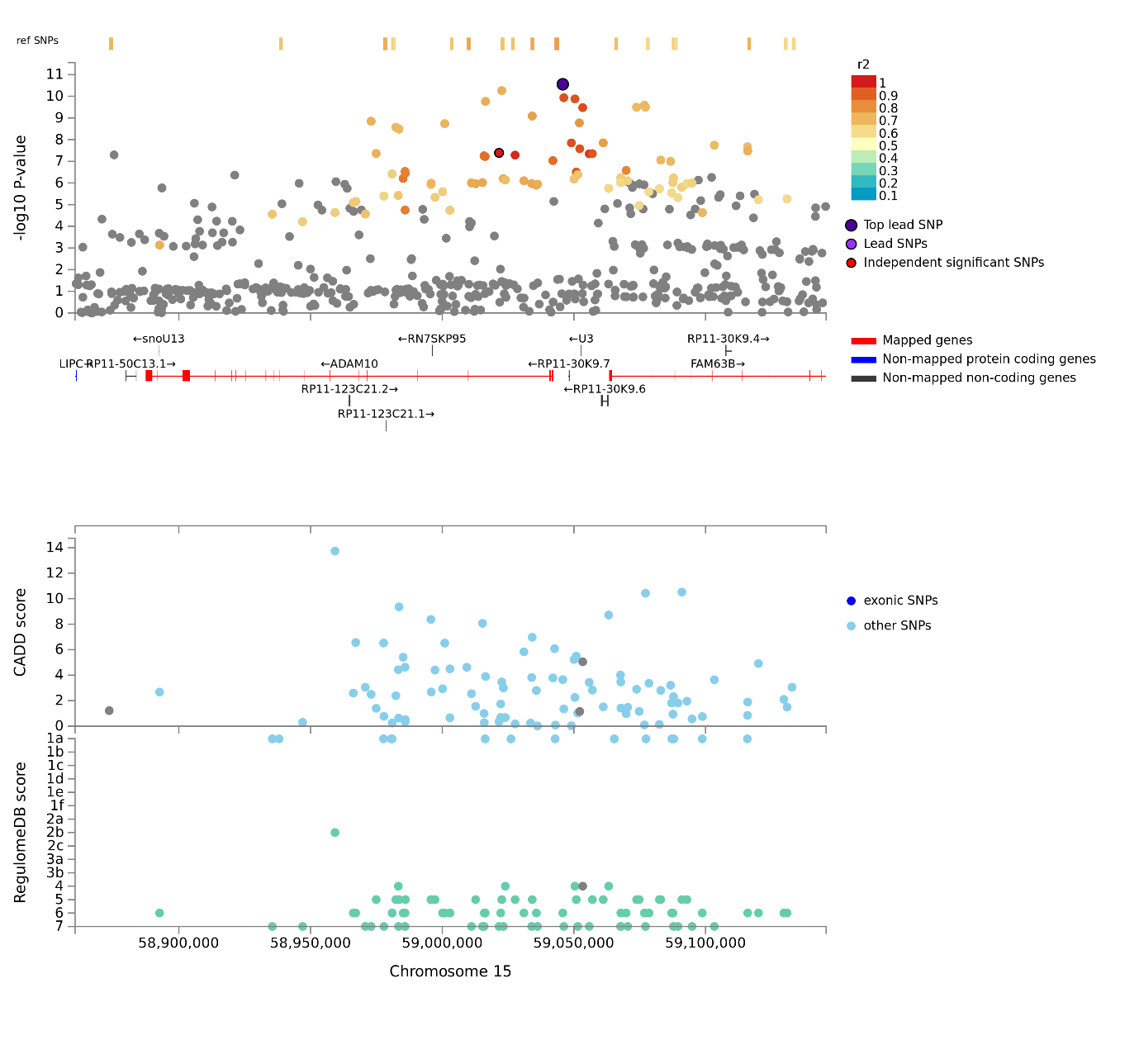


**Figure S2.** Plot of genome-wide significant *BCKDK* locus


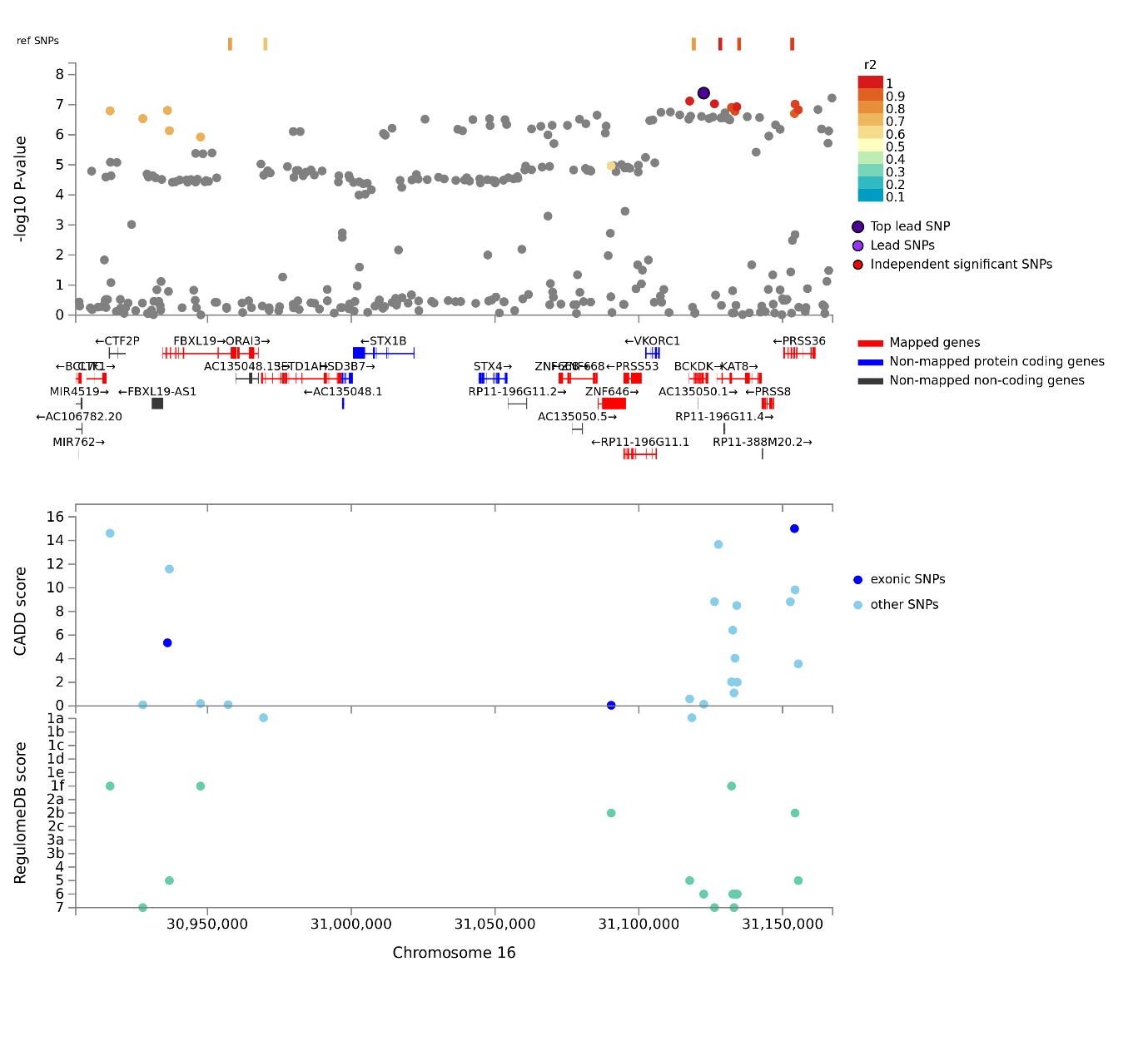


**Figure S3.** Plot of genome-wide significant *ACE* locus


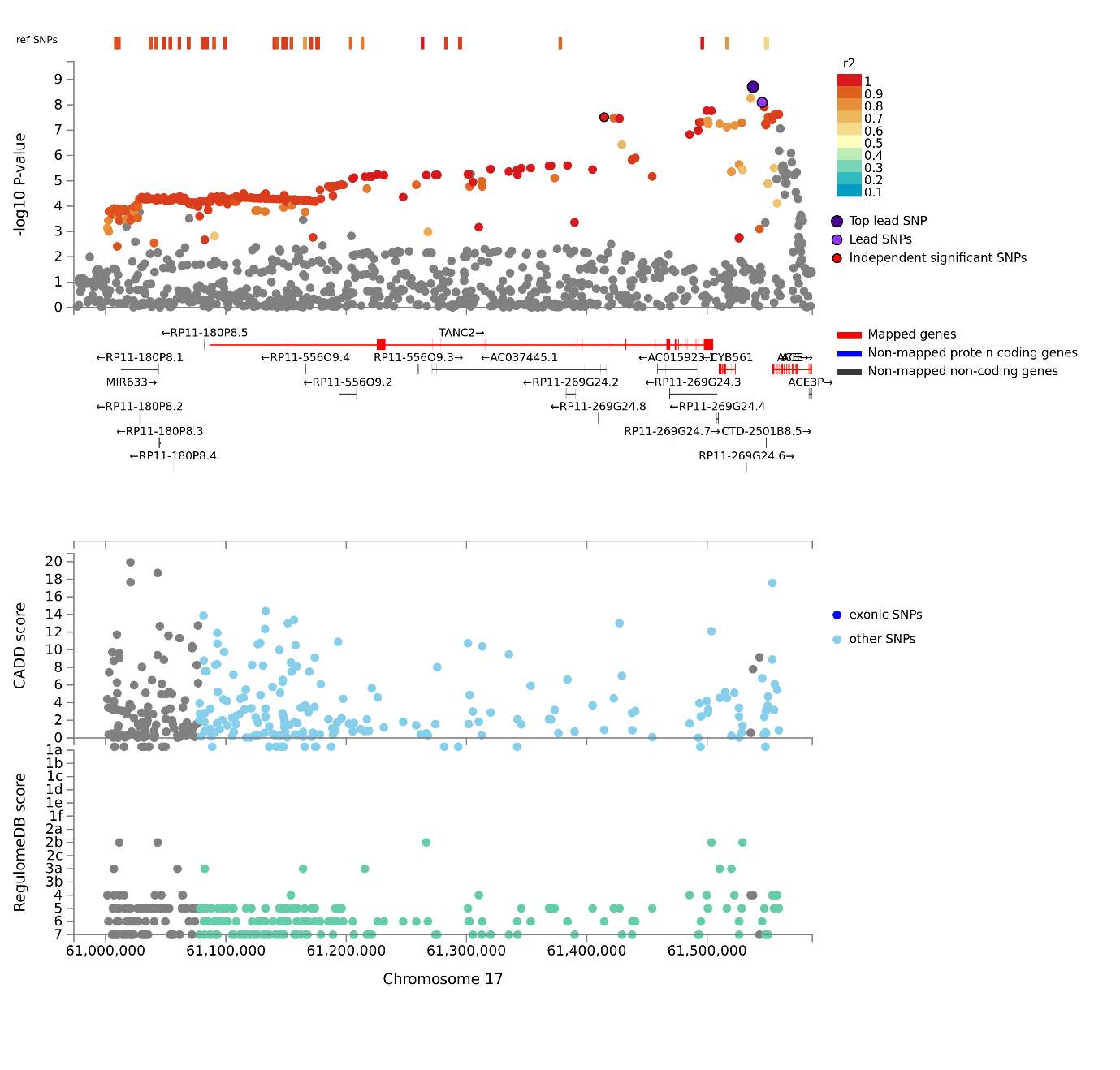


**Figure S4.** SMR and HEIDI plot of *KAT8* (eQTL analysis)


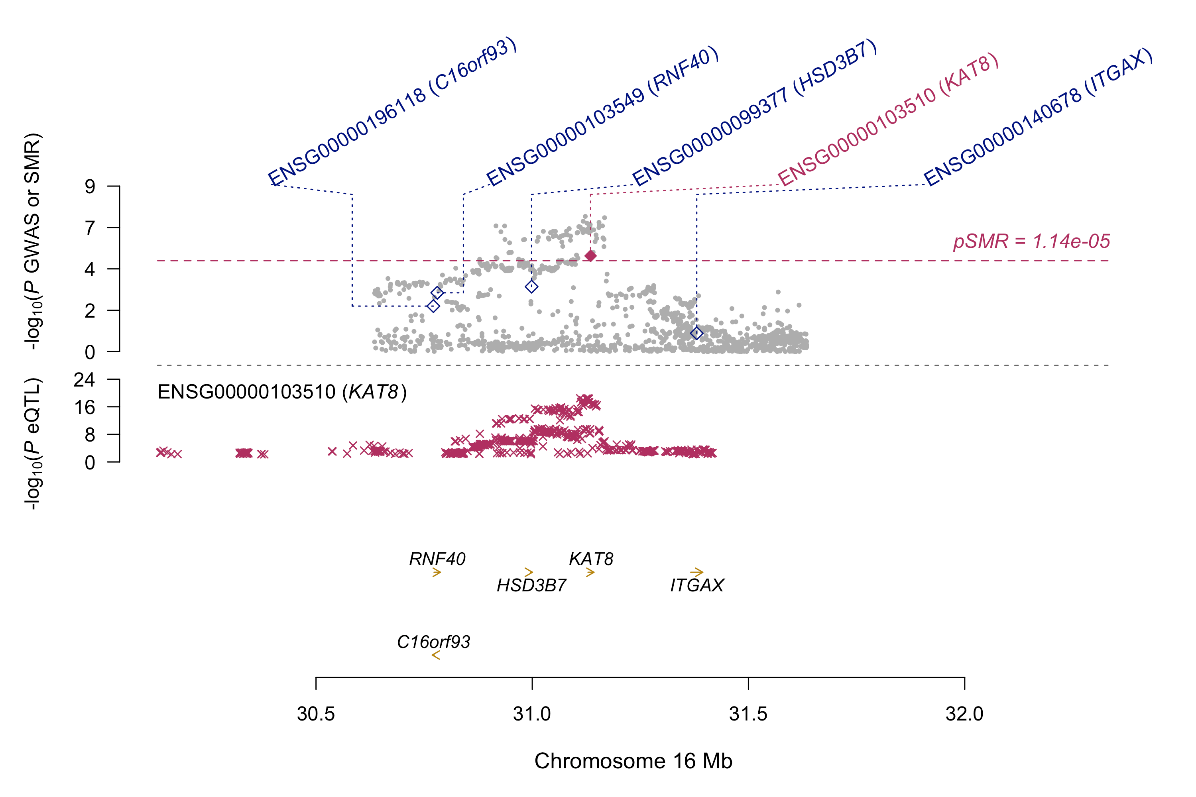


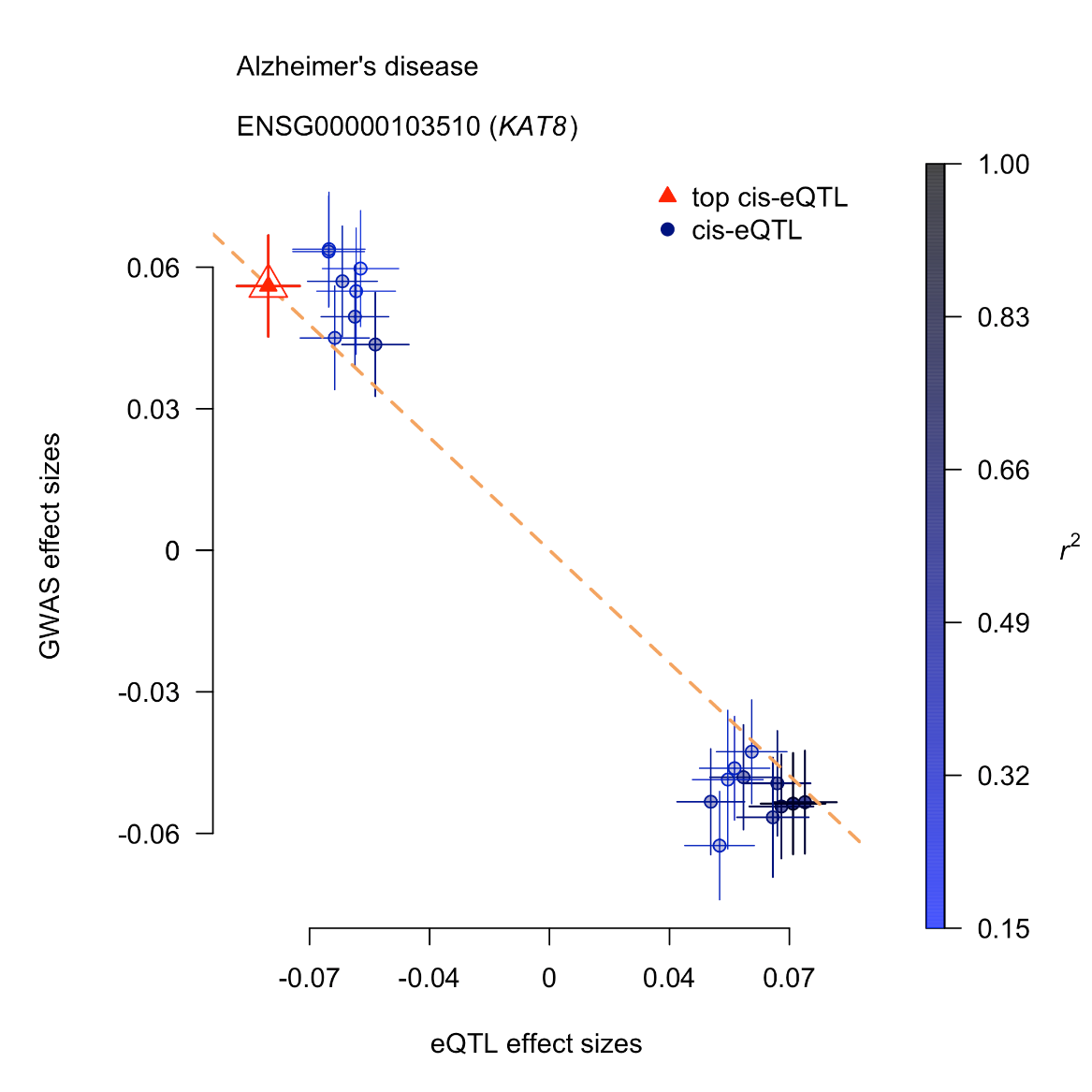


**Figure S5.** SMR and HEIDI plot of *CR1* (eQTL analysis)


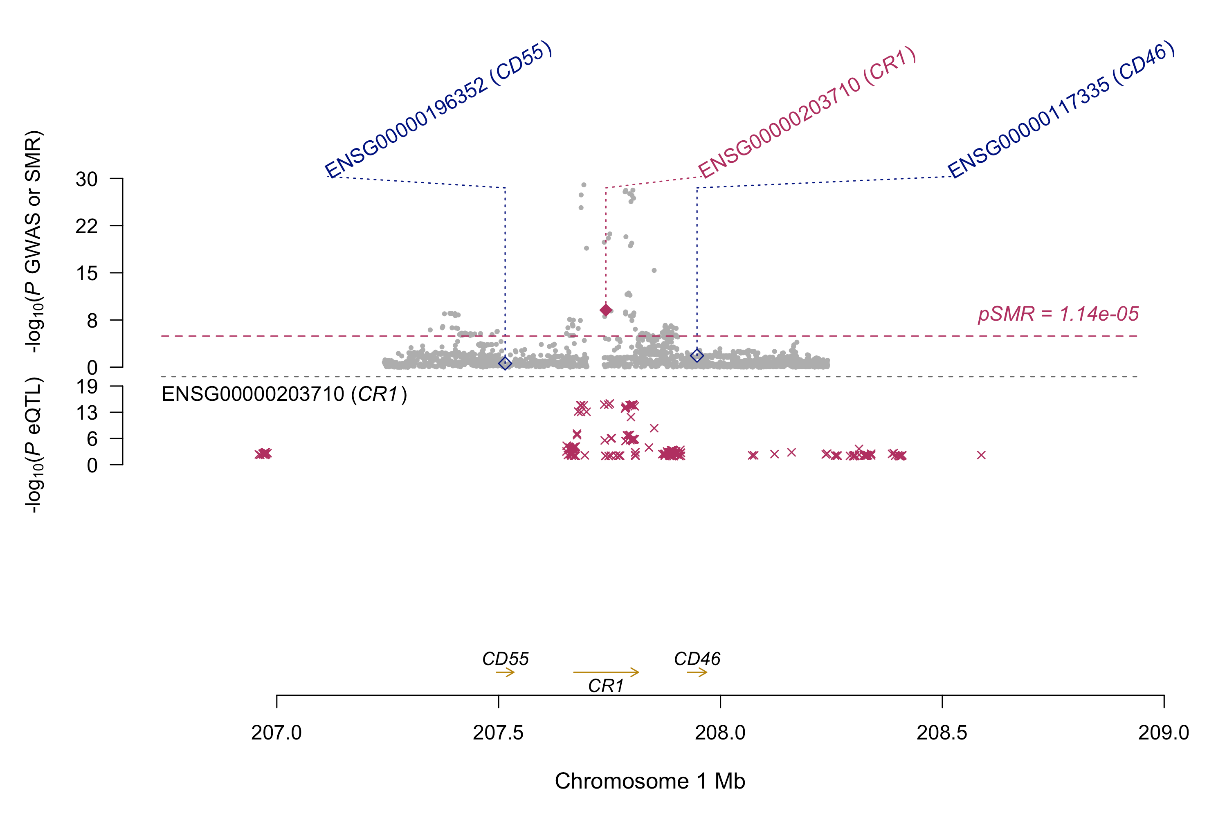


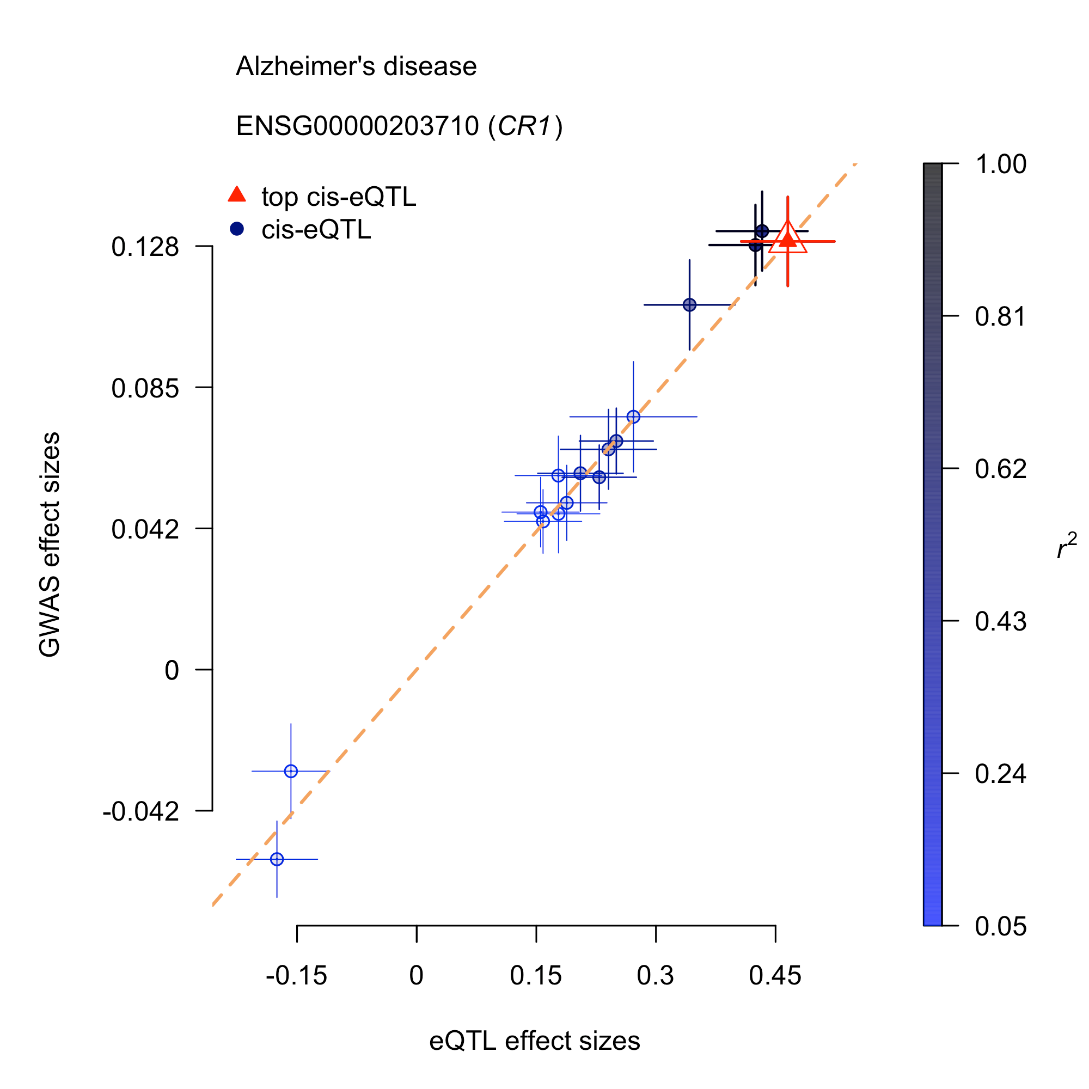


**Figure S6.** SMR and HEIDI plot of *STAG3* (methQTL analysis)


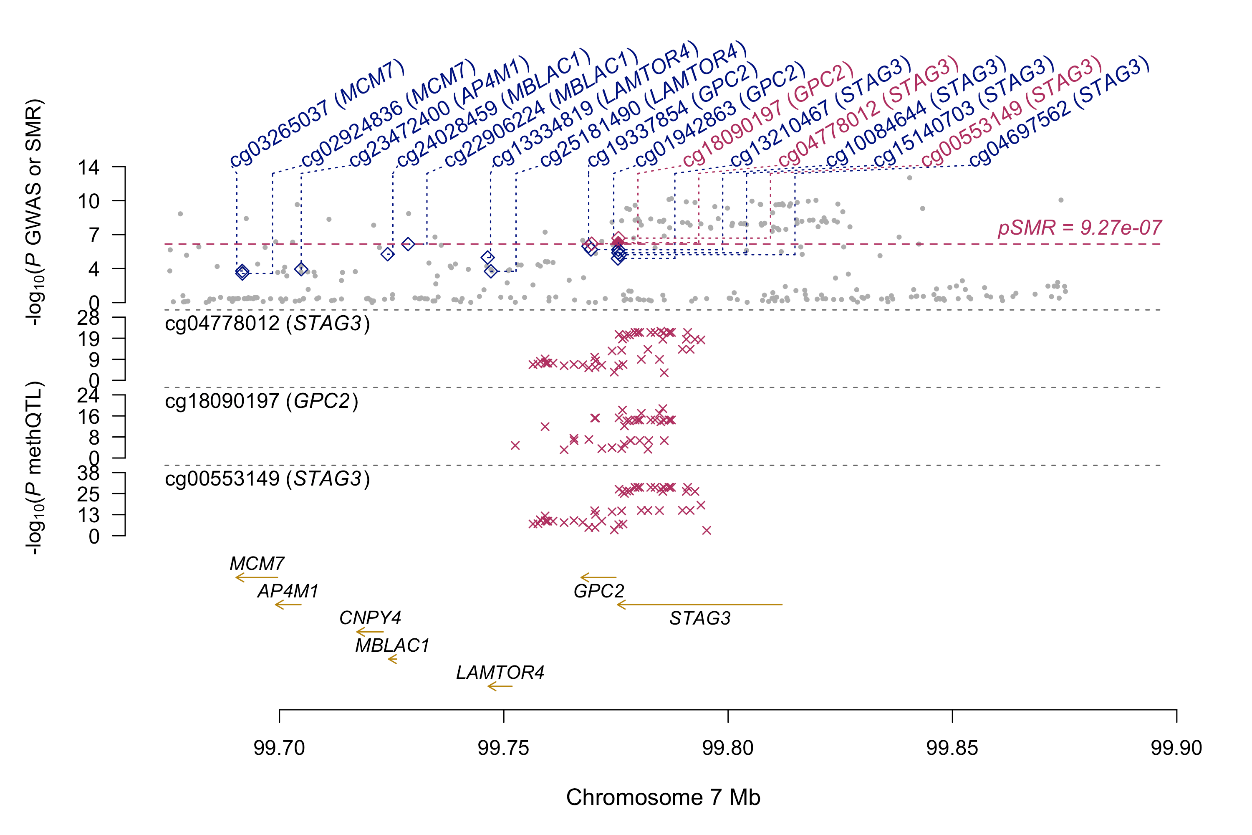

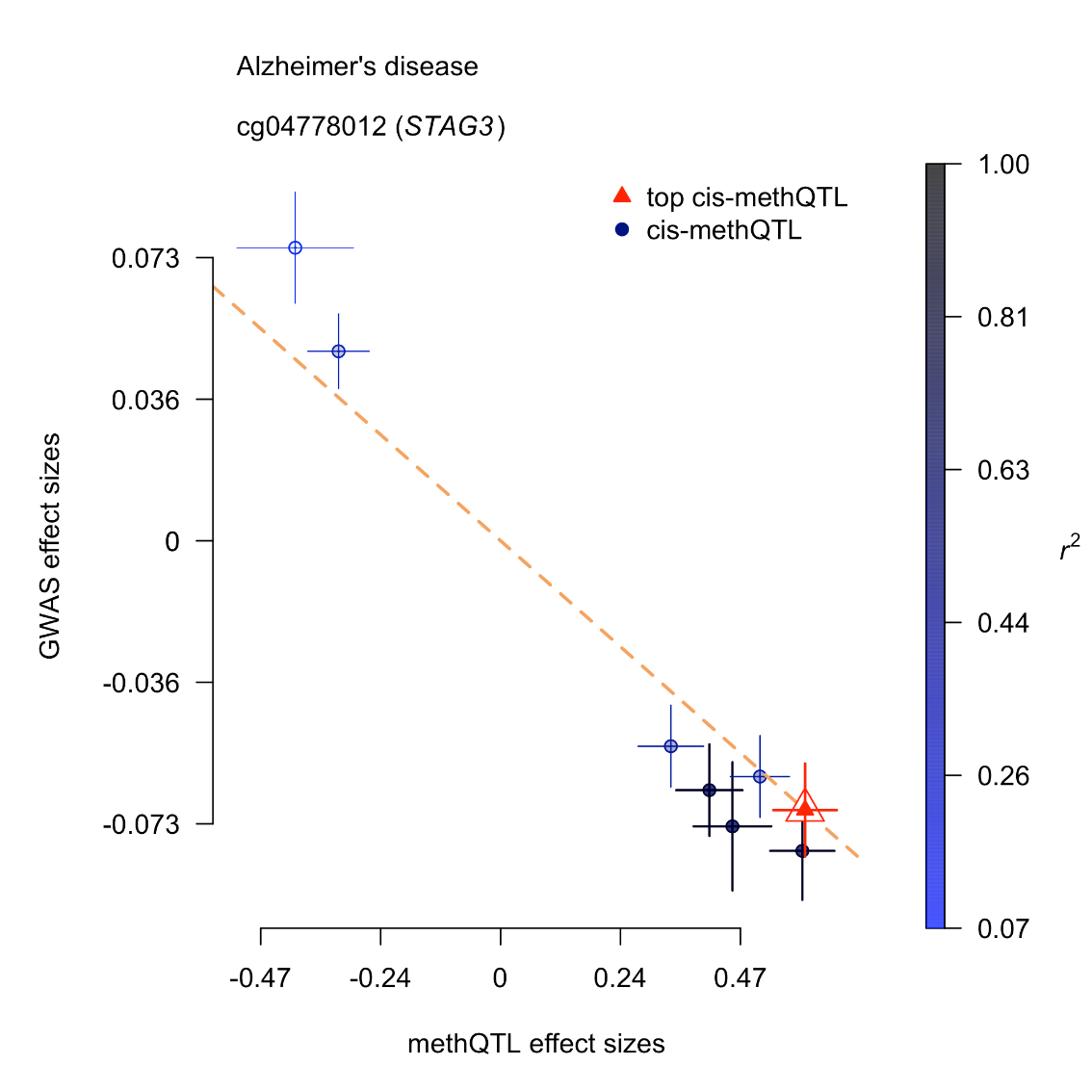


**Figure S7.** SMR and HEIDI plot of *CD2AP* (methQTL analysis)


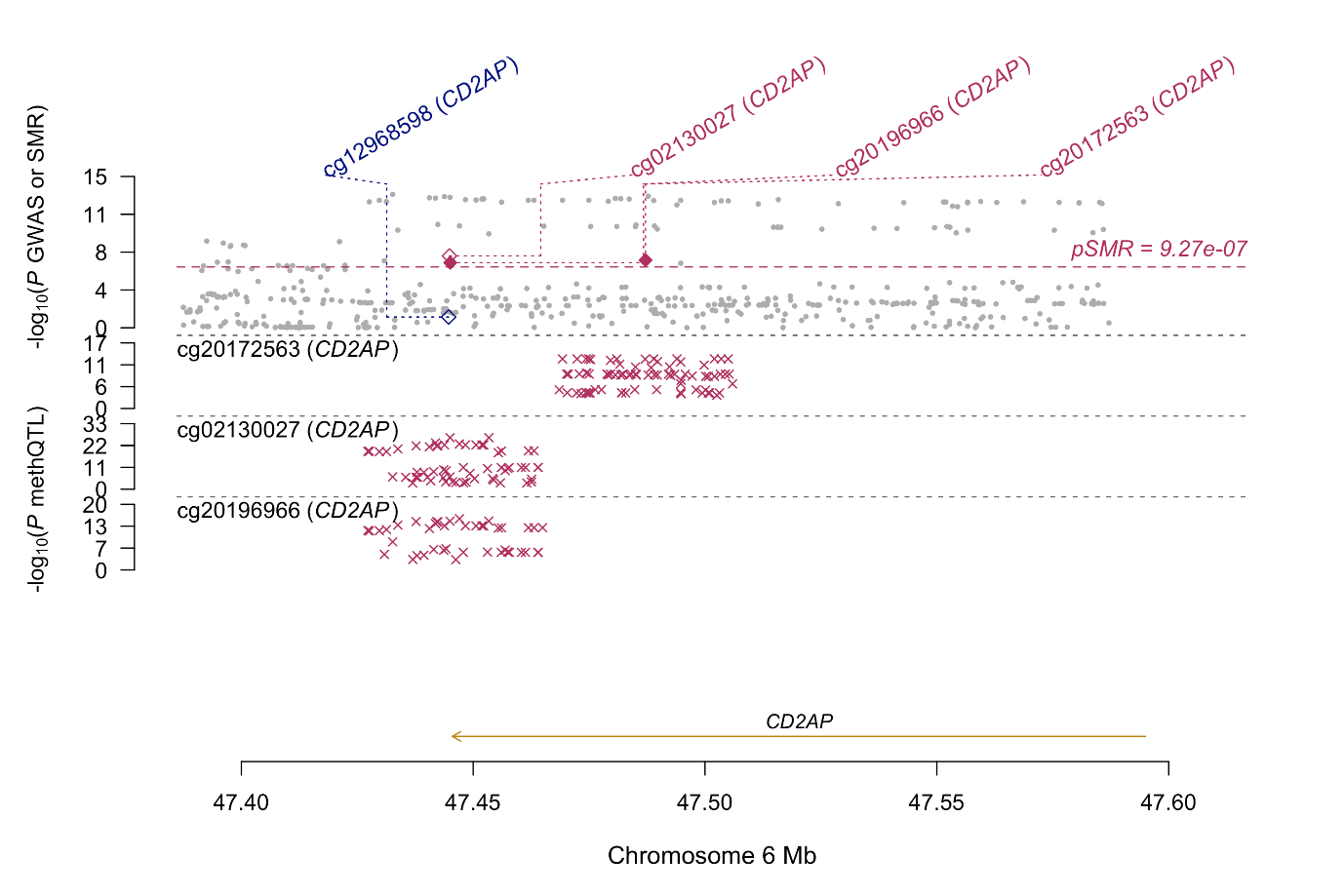

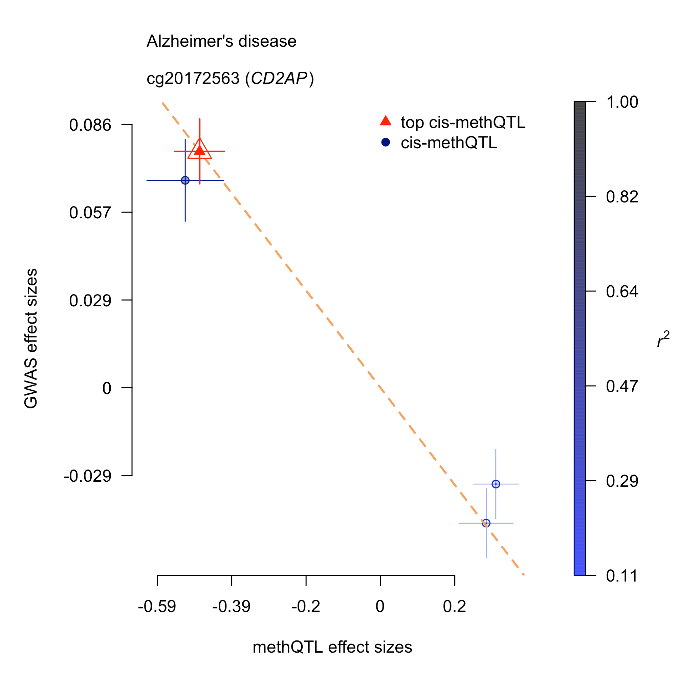

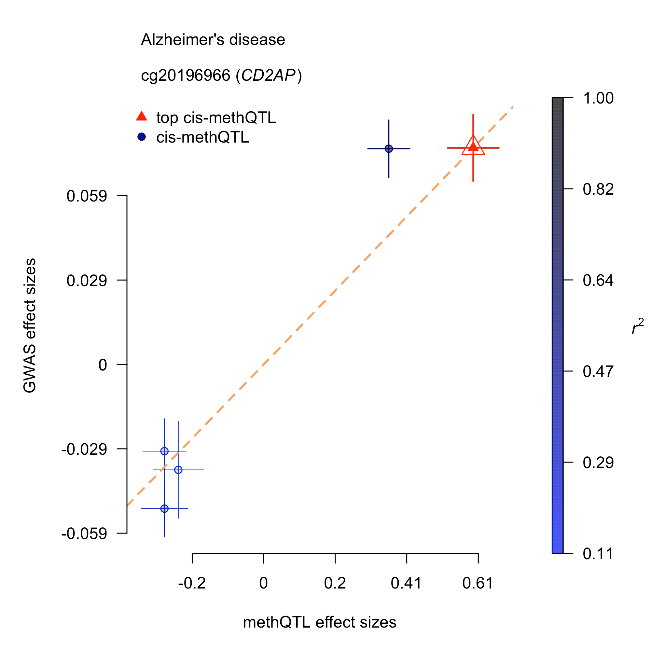


**Figure S8.** SMR and HEIDI plot of *HLA-DRB1* (methQTL analysis)


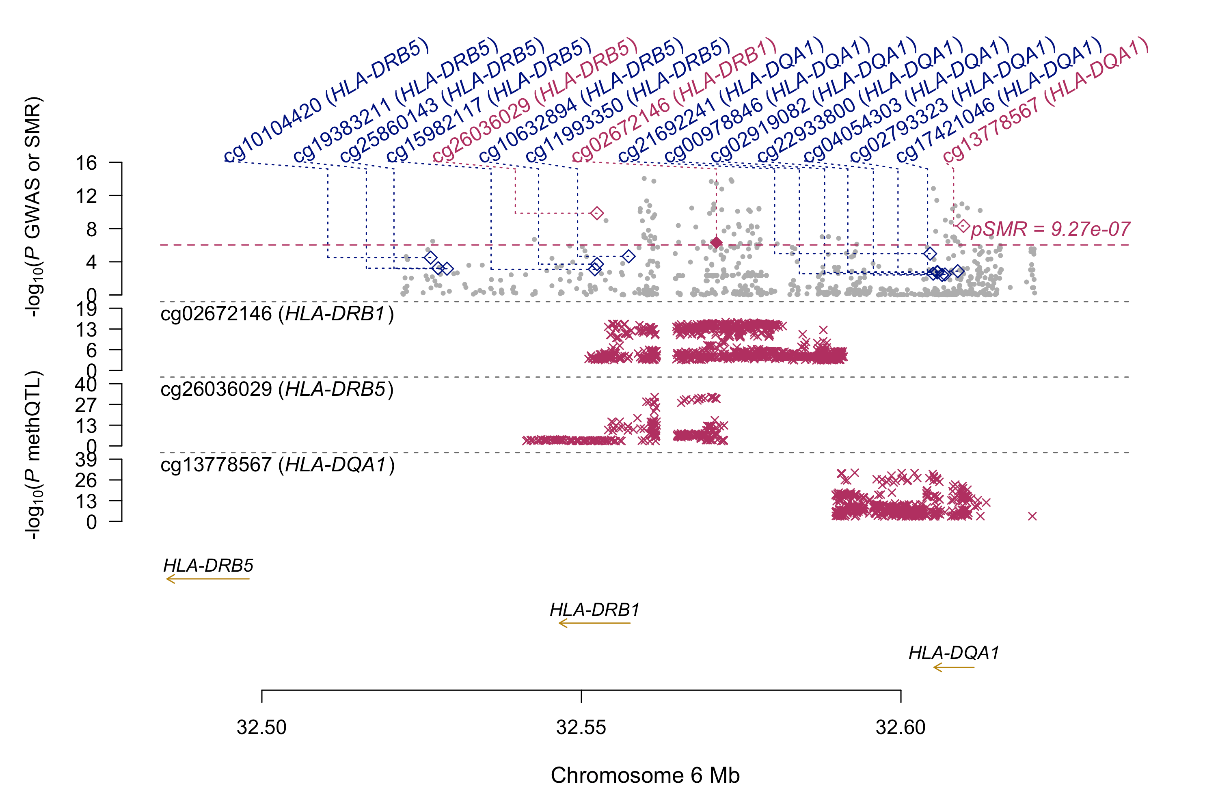


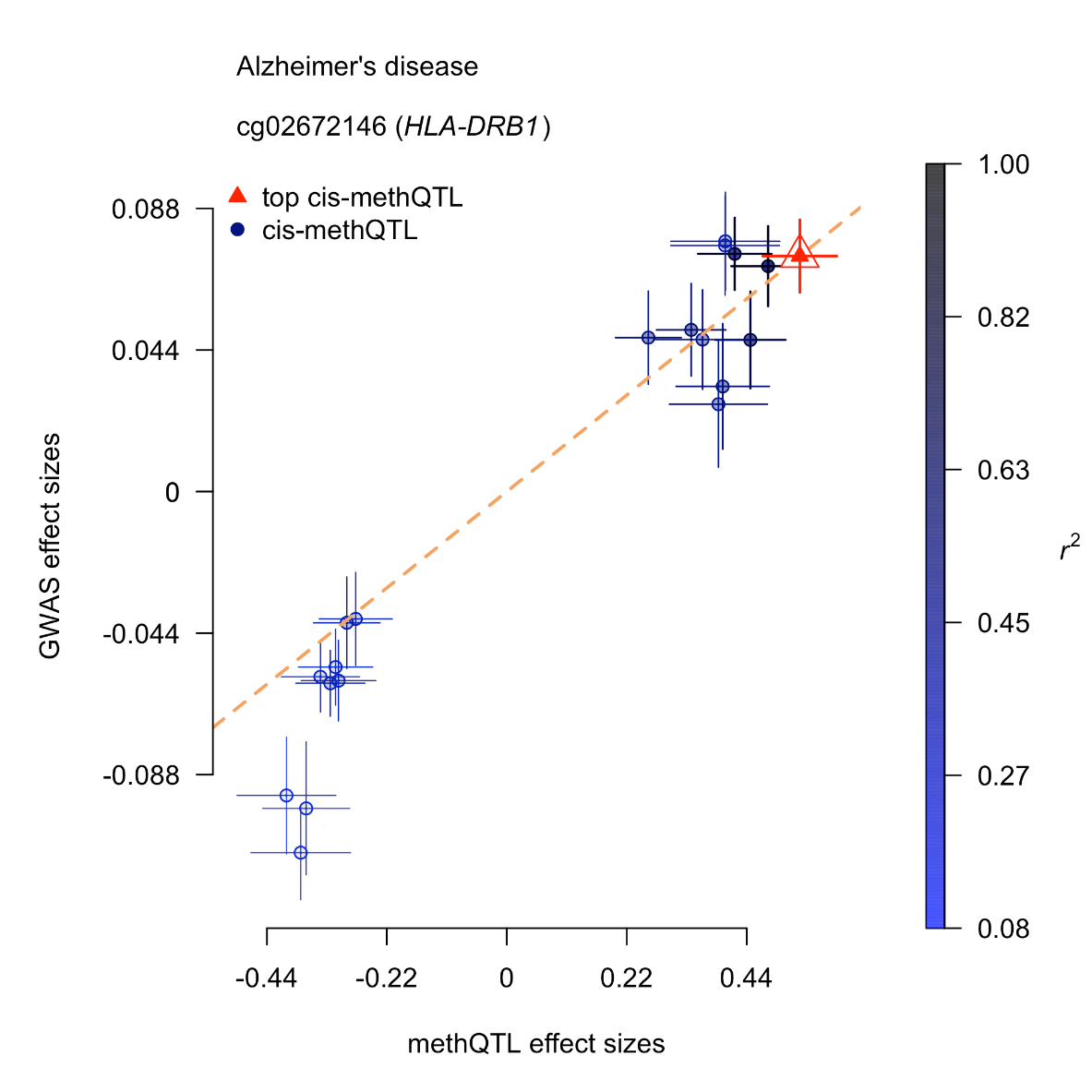


**Figure S9.** SMR and HEIDI plot of *PSMC3* (methQTL analysis)


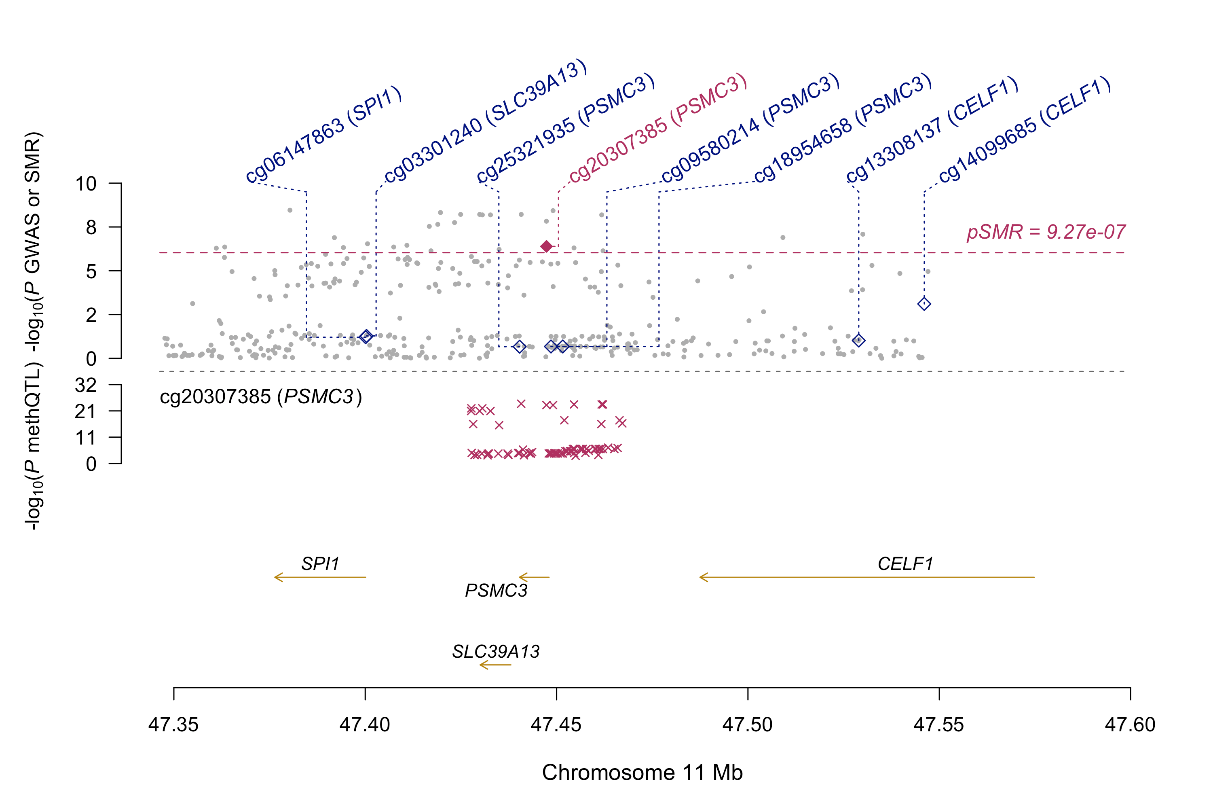


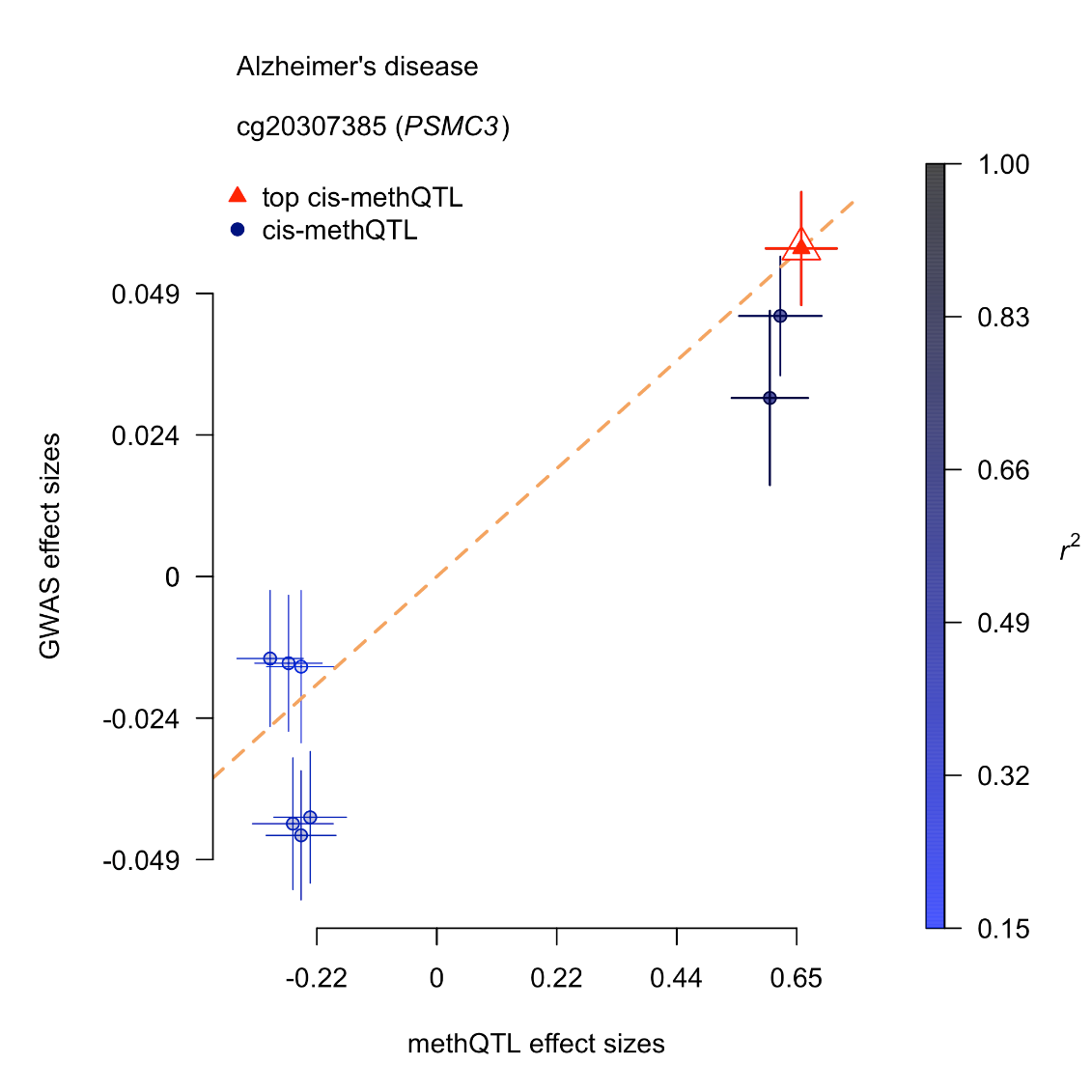

Supplement: Supplementary file 1 — Supplementary Figures [file 41398_2018_150_MOESM1_ESM.docx]
