# Supplementary material for: GWAS on family history of Alzheimer’s disease
Source: Transl Psychiatry. 2018 May 18;8:99. doi: 10.1038/s41398-018-0150-6 (PMC5959890; doi:10.1038/s41398-018-0150-6)
Supplement: Supplementary file 3 — Supplementary Note [file 41398_2018_150_MOESM3_ESM.docx]

**Supplementary Note 1.** Calculation of standard error from an odds ratio and p-value.

From the statistical theory for an association test

$$Z = \frac{\beta}{\mathrm{SE}}$$

where Z is the test statistic, β is the regression coefficient with standard error, SE

A p-value for the regression coefficient, β, for a two-tailed test is calculated by

$$P= 2 \times\Phi(-\left| Z \right|)$$

To back-calculate the SE from P and β we therefore use the formula

$$SE=\left| \frac{\beta}{\Phi^{-1}(\frac{P}{2})} \right|$$
